# Number of Chronic Medical Conditions and Quality of Life of Ethnic Minority Older Adults

**DOI:** 10.3390/geriatrics7050106

**Published:** 2022-09-29

**Authors:** Sharon Cobb, Babak Najand, Tara Gravidez, Berlin Navarro, Alondra Herreraramos, Mohsen Bazargan

**Affiliations:** 1 Mervyn M. Dymally School of Nursing, Charles R Drew University of Medicine and Science, Los Angeles, CA 90059, USA; 2 Marginalization-Related Diminished Returns (MDRs) Research Center, Los Angeles, CA 90059, USA; 3 Department of Family Medicine, Charles R Drew University of Medicine and Science, Los Angeles, CA 90059, USA; 4 Department of Urban Public Health, Charles R Drew University of Medicine and Science, Los Angeles, CA 90059, USA; 5 Department of Family Medicine, University of California Los Angeles (UCLA), Los Angeles, CA 90095, USA

**Keywords:** multimorbidity, chronic disease, quality of life, population groups

## Abstract

Background: The Blacks’ mental health paradox is defined as disproportionately better mental health among Black individuals compared to White individuals, despite their higher exposure to a wide range of adversities. However, the existing literature on this phenomenon is mainly limited to studies that have compared Black and White individuals. There has been little research on this phenomenon among ethnic groups other than Whites. Objectives: This study tested the Blacks’ mental health paradox with consideration of Latinx individuals as the control group. Methods: This cross-sectional study collected demographic data, socioeconomic status, chronic medical conditions, and mental and physical quality of life of 724 older Black and Latinx adults residing in low socioeconomic areas of south Los Angeles. Linear regressions were used for data analysis with mental and physical health-related quality of life (HRQoL) as dependent variables and the number of chronic medical conditions as the independent variable. Results: Overall, a higher number of chronic medical conditions was associated with lower mental and physical quality of life. A statistically significant interaction was found between race/ethnicity and the effect of the number of chronic medical conditions on mental HRQoL, which was indicative of Blacks’ mental health paradox. Conclusion: Older Black adults with a higher number of chronic medical conditions report better mental health compared to their Latinx peers with the same number of chronic medical conditions. Thus, Blacks’ mental health paradox can be seen when Black and Latinx populations are compared. Replication of such a paradox provides additional support for the relative mental health advantage of Black people compared to other ethnic groups.

## 1. Background

Poor physical health (e.g., multimorbidity) is associated with lower health-related quality of life (HRQoL) [1,2]. Individuals with specific types of chronic medical conditions, such as heart disease, cancer, asthma, and arthritis, are more likely to be depressed and report lower health-related quality of life (HRQoL) [3]. Several national and local studies in community [3] and clinical [4] settings in adults and older adults have shown that a high number of chronic medical conditions is a risk factor for poor mental health and well-being (e.g., HRQoL) [3].

The association between the number of chronic medical conditions and poorer mental well-being [5,6,7] may, however, vary by race/ethnicity [8,9,10,11,12,13,14,15]. This is in part because different populations utilize various coping mechanisms to deal with adversities, as some groups may have higher preparedness to face a new adversity or access to resources (e.g., healthcare) [16]. Some studies suggest that the link between chronic medical conditions and mental well-being may be weaker for Black individuals compared to White individuals [8,9,10,11,12,13,14,15]. However, most available comparative studies have exclusively compared Black and White individuals. Thus, we are unaware of any comparative studies of Black people that have included a non-White control group (e.g., Latinx).

The Blacks’ mental health paradox refers to the superior mental well-being of Black populations, despite their higher prevalence of adversities and chronic medical conditions. This phenomenon reflects the relative resilience of Black populations, and particularly of older adults. Various scholars have attributed this observation to growth and flourishing in the presence of adversity. However, it is unknown whether this paradox will hold true if we compare Black individuals with other ethnic groups (other than Whites). In a low-income community with majority Black and Latinx populations [17,18,19], the current study tested the Blacks’ mental health paradox with inclusion of Latinx individuals as the control group. Replication of the Blacks’ mental health paradox with Latinx individuals as a control group would suggest that Blacks’ mental health paradox is a stable and robust phenomenon which holds regardless of the control group (at least for Latinx individuals).

## 2. Methods

This cross-sectional study was conducted between the years 2015 to 2020 in areas of low socioeconomic status in South Los Angeles. Participants were recruited from low-income housing, churches, and other community venues within this communal environment. Participants were sampled in a non-randomized setting and all participants were individually interviewed with a healthcare provider in their location, which was convenient and maintained confidentiality. All participants resided in Service Planning Area 6 (SPA 6) of South Los Angeles, which is one of the most impoverished areas of Los Angeles, with most residents identifying as Black or Latinx [20]. In SPA6, 61% were Latino, 34% were Black, 3% were White and 2% were Asian/Other [19]. All participants had at least one chronic medical condition and were aged sixty-five or older. For more information on the methodology of this study, please see our previous publications [21,22,23,24,25]. The sample size included 724 individuals (116 Latinx and 608 Black). 

This study collected data on demographic factors, including race/ethnicity, age, sex/gender, socioeconomic status (education, etc.), living condition, insurance status, physical health, and mental and physical HRQoL. A Latinx interviewer, who was MD MPH and bilingual, conducted the interviews. The 12-Item Short Form Survey (SF-12) assessed mental and physical HRQoL [26,27]. The SF-12 is one of the most widely used measures of quality of life. It has high validity and reliability with low burden to the patients and individuals, which reduces their fatigue [28]. Thus, the SF-12 generates mental and physical quality of life scores, with higher scores reflecting better well-being [29,30]. Chronic medical conditions (CMCs) included: (1) stomach or intestinal problems; (2) asthma or bronchitis; (3) arthritis; (4) hypertension/high blood pressure; (5) heart diseases; (6) diabetes; (7) chronic back pain; (8) cancer; (9) endocrine conditions and thyroid problems; (10) stroke; and (11) migraine/headache. The number of CMCs was measured by asking whether participants have been diagnosed with the conditions listed above [21,22,23,24,25].

## 3. Data Analysis

Data analysis was performed using the Statistical Package for Social Sciences (SPSS), version 22 (SPSS Inc., Chicago, IL, USA). In order, univariate, bivariate, and multivariable statistical methods were used for data analysis. For univariate analysis, we reported frequencies (percentages) for categorical variables and means and standard deviations for continuous measures. For bivariate analysis, chi-square tests and independent samples *t*-tests were employed in order to explore associations between race/ethnicity and study variables. Finally, we used linear regressions to test the overall association between our independent variable, namely, the number of chronic medical conditions, and our outcomes, namely, physical or mental quality of life. All confounders were controlled in our linear regression model. We used the “Enter” rather than the “Forward” or “Backward” method, meaning that independent and confounding variables remained in the model even if they were not statistically significant. As both independent and dependent variables were continuous, a beta was our measure of effect size, and a negative number was indicative of the effect of number of chronic medical conditions on physical or mental quality of life. In order to test racial/ethnic variation in this association, we included an interaction term, which was the multiplicative effect of ethnicity and the number of chronic medical conditions. Latinx was coded as one and non-Latinx as zero.

## 4. Results

This study included 724 individuals, with 116 identifying as Latinx and 608 as Black. Table 1 shows the results of univariate analysis overall and by ethnicity. This table also shows differences between ethnic groups in terms of demographic data, SES, number of chronic medical conditions, and physical and mental quality of life. Compared to Latinx participants, Black participants had higher ages and education levels, better HRQoL, and a higher number of chronic medical conditions. The groups did not differ in gender or insurance.

Table 2 shows the overall bivariate correlation. This table shows an inverse association between the number of chronic medical conditions and physical and mental HRQoL.

Table 3 and Table 4 show the linear regression results with physical or mental HRQoL as the outcome, demographic data and SES as covariates, and the number of chronic medical conditions as the independent variable. These models are employed overall in both the absence and presence of ethnicity by the interaction term, which was the number of chronic medical conditions. Overall, a higher number of chronic medical conditions was associated with lower mental and physical HRQoL of participants in the overall population. We documented a statistically significant interaction between race/ethnicity and the number of chronic medical conditions on mental, but not physical, HRQoL. 

## 5. Discussion

This study revealed an inverse association between a higher number of chronic medical conditions and worse physical and mental HRQoL. While the association between the number of chronic medical conditions and physical HRQoL was similar in Black and Latinx individuals, we found an interaction which was suggestive of Blacks’ mental health paradox, which is defined as better mental well-being among the Black population despite their overall higher number of chronic medical conditions [8,9,10,11,12,13,14,15].

The finding that a higher number of chronic medical conditions is associated with worse physical and mental HRQoL is well-documented, as mental and physical aspects of health tend to covary [5,6,7]. Moreover, being diagnosed with a chronic condition lowers physical and mental HRQoL, regardless of the type of condition [5,6,7]. This association is reported for many chronic diseases, including but not limited to cardiovascular [31], renal [14], gastrointestinal [32], and respiratory conditions [33]. These chronic conditions impose limitations on activities of daily living [34], and may cause depression [35] and disability [36]. Additionally, chronic conditions may cause fatigue [37], pain [38], and other disabling symptoms [39]. Fear of death may impact well-being of individuals with medical conditions [40]. Other aspects of well-being, such as sleep, sexuality, mobility, and socialization, which all have inverse effects on individuals’ HRQoL, may also be limited [41]. As a result, the number of chronic medical conditions an individual has, regardless of their type or severity, is a risk factor for low HRQoL. Across various chronic conditions, however, chronic renal disease may have the highest impact on depression, and cardiovascular conditions may have the highest impact on anxiety [2].

As shown in this study, the Blacks’ mental health paradox holds even when we compare Black and Latinx individuals. This study is unique because it extends what we know from Black–White differences to more diverse ethnic groups. This is an interesting finding, as the Blacks’ mental health paradox has been validated frequently, but only for comparison of Black and White controls [42,43,44]. As all of these studies have only recruited Black and White individuals [8,9,10,11,12,13,14,15], less is known about any similar differences between Black and Latinx individuals. Despite chronic medical conditions being risk factors for poor mental well-being, Black individuals with chronic conditions report better mental HRQoL. This means that chronic medical conditions do not similarly result in the same degree of poor mental well-being for Black, White, and Latinx individuals [8,9,10,11,12,13,14,15]. As the impact of the number of chronic medical conditions on mental quality of life is greater for Latinx individuals, Black individuals with multiple chronic medical conditions maintain good mental well-being. The same, however, cannot be said for physical quality of life.

As mentioned before, this phenomenon has already been shown for Black–White comparisons [8,9,10,11,12,13,14,15]. In cross-sectional and longitudinal studies, associations between the number of chronic medical conditions and poor mental health are shown to be stronger for White individuals than Black individuals [8,9,10,11,12,13,14,15]. In one study that followed participants for 25 years, bidirectional effects of depression and chronic disease and disability were mainly present for White but absent for Black individuals [45]. In another study that also followed participants for 25 years, emergence of the Black–White mental and physical health paradox was observed over time [17]. As these comparative studies have only recruited Black and White individuals, we were not aware of any comparative study of Black and other non-White control group. This is why this study makes a unique and major contribution to the literature.

We propose three explanations for our observed racial/ethnic variation. First, this phenomenon can be interpreted as a relative resilience of Black populations, particularly older Black adults, compared to other groups. Keyes and colleagues [18] have argued that this phenomenon may reflect preparedness of Black people in facing adversity. Mezuk, Jackson, and colleagues [16] argue that Black individuals are more prepared than other groups to negate some effects of depression by lowering their stress through behavioral risks such as consuming an unhealthy diet [46]. Jackson and Mezuk [16] have proposed than Black people may use some unhealthy behaviors to maintain their mental health; however, some contrasting results are also shown [47]. It may be inferred that Black individuals, who have faced long histories of institutionalized and structural racism and discrimination, have learned to cope with adversity and access to fewer resources [48,49]. The second explanation for this phenomenon is that the mental wellness of Black individuals may imply a smaller effect of chronic diseases, due to the larger impact of of sociodemographic factors and other adversities [50]. This aligns with the theory of minorities’ diminished returns, suggesting that similar resources may not result in similar outcomes for marginalized and privileged groups, because marginalized people face more discrimination, stress, and racism in their lives [51,52,53]. In other words, racism, discrimination, stress, and low SES may be detrimental for Black people, regardless of their chronic disease status [51,52,53]. The third explanation is that due to racism and discrimination, presence or absence of resources and assets may have lost some of their protective effects. For example, due to other adverse conditions, lack of physical health conditions may not be enough to enhance the sense of wellbeing of Black individuals [54,55,56,57]. The fourth explanation suggests that Black individuals may better utilize religion, spirituality, and social support than other groups [58,59]. For example, some research has shown stronger protection of religion and social support among Black people [60,61,62,63,64,65,66]. Some other studies by Mouzon and colleagues, however, have failed to show a full explanation of the mechanisms for such a paradox [67,68]. Religion and spirituality are shown to be very beneficial for Black people.

The results have clinical implications. At any level of mental HRQoL, ethnic groups have different physical health statuses. This information is relevant to the provision of integration of physical and mental health care for ethnic minorities. In addition, clinicians need to monitor for chronic disease in Black patients regardless of their well-being. For Latinx patients, however, there is an increasing need for monitoring and screening of chronic diseases when HRQoL is low.

Study limitations include non-randomized sample size, low sample size of Latinx group, self-reported data on chronic medical conditions, lack of analysis of each type of chronic disease, and omission of variables that may confound our association of interest. Given the small sample size and non-random sampling, the results are not able to be generalized to apply to older American adults or even our SPA6 population. This was a preliminary and local study with convenient sampling, and there is a need for future research with a random, large U.S. sample. Black and Latinx sample sizes were also unequal, so the results may be biased due to a higher number of Black than Latinx individuals. Factors such as year of diagnosis, medications used, and adherence to medications may have confounded our association of interest. Thus, our study had unmeasured confounders. Finally, although self-reported data are commonly used to collect information on chronic diseases, the validity of the results would be increased if we could confirm those conditions through medical records. Given these limitations, the results should be interpreted with caution. Thus, there is a need for future quantitative and qualitative research on this topic. Qualitative methods may provide very relevant information on Black individuals’ resilience and mental health advantages.

## 6. Conclusions

Despite the inverse association that exists between number of chronic medical conditions and poor physical and mental HRQoL, the link between the number of chronic medical conditions and poor mental HRQoL is weaker for Black than Latinx individuals. This is similar to and supports the Blacks’ mental health paradox, in which Black individuals report better mental health despite higher prevalence of adversities and chronic medical conditions. This pattern is not merely limited to the comparison of Black and White people, as evidenced supported by the study, it may hold for comparison of Black individuals with other similarly under-resourced minority groups such as Latinx population.

## Figures and Tables

**Table 1:** Descriptive data overall and by race/ethnicity.

	All *n* = 724		Latinx *n* = 116		Black *n* = 608		
	*n*	%	*n*	%	*n*	%	
Gender							
Male	252	34.8	40	34.5	212	34.9	NS
Female	472	65.2	76	65.5	396	65.1	
Marital Status							
Not Married	585	80.8	74	63.8	511	84.0	*
Married	139	19.2	42	36.2	97	16.0	
Medicare Insurance							
No	198	27.3	31	26.7	167	27.5	NS
Yes	526	72.7	85	73.3	441	72.5	
	Mean	SD	Mean	SD	Mean	SD	
Age	73.86	6.894	73.32	6.443	73.96	6.977	*
Years of Education	11.91	3.275	7.74	4.263	12.70	2.320	*
Number of Chronic Conditions	4.0870	2.02458	3.9569	2.14838	4.1118	2.00099	*
Mental HRQoL SF12	53.0375	10.65799	50.6646	14.57613	53.4902	9.68333	*
Physical HRQoL—SF12	40.9067	11.97457	39.6297	11.55544	41.1503	12.04674	*

* *p* < 0.05 for comparison of Latinx and Black, HRQoL: Health-Related Quality of Life, NS: Non-significant.

**Table 2:** Correlation between study variables overall.

	1	2	3	4	5	6	7	8	9	10
1 Age	1	−0.139 **	0.034	0.044	−0.079 *	0.112 **	0.018	−0.004	0.044	0.065
2 Years of Education		1	0.556 **	0.089 *	−0.043	0.078 *	−0.077 *	−0.046	0.136 **	0.047
3 Black			1	−0.003	−0.189 **	0.188 **	−0.006	0.028	0.097 **	0.047
4 Gender				1	−0.152 **	0.074 *	−0.032	0.123 **	0.046	−0.152 **
5 Married					1	−0.442 **	0.000	−0.049	0.016	0.089 *
6 Living Arrangement						1	−0.022	0.107 **	−0.068	−0.135 **
7 Medicare							1	0.034	0.004	−0.033
8 Chronic Diseases (*n*)								1	−0.232 **	−0.464 **
9 Mental HRQoL—SF12									1	−0.025
10 Physical HRQoL—SF12										1

HRQoL: Health-Related Quality of Life,* *p* < 0.05, ** *p* < 0.01.

**Table 3:** Linear regression on mental health-related quality of life as the outcome.

	Model 1						Model 2					
	Unstand b	SE	Beta	95% CI		*p*	Unstand b	SE	Beta	95% CI		*p*
Black	1.721	1.301	0.059	−0.833	4.275	0.186	−3.339	2.351	−0.115	−7.954	1.275	0.156
Age	0.094	0.057	0.061	−0.018	0.205	0.100	0.089	0.057	0.058	−0.022	0.200	0.117
Gender	1.560	0.825	0.070	−0.059	3.179	0.059	1.606	0.822	0.072	−0.007	3.219	0.051
Years of Education	0.333	0.145	0.102	0.048	0.619	0.022	0.336	0.145	0.103	0.051	0.620	0.021
Married	0.106	1.101	0.004	−2.056	2.268	0.924	0.036	1.097	0.001	−2.119	2.190	0.974
Living Arrangement	−1.540	0.869	−0.072	−3.247	0.166	0.077	−1.549	0.866	−0.072	−3.249	0.151	0.074
Medicare Coverage	0.470	0.860	0.020	−1.218	2.157	0.585	0.445	0.856	0.019	−1.236	2.126	0.604
Chronic with no depression	−1.211	0.192	−0.230	−1.587	−0.834	0.000	−2.250	0.446	−0.427	−3.125	−1.375	0.000
Chronic with no depression × race/ethnicity							1.265	0.490	0.282	0.302	2.227	0.010

Model 1: main effects, Model 2: main effects and interaction.

**Table 4:** Linear regression with physical quality of life as the outcome.

	Model 1						Model 2					
	Unstand b	SE	Beta	95% CI		*p*	Unstand b	SE	Beta	95% CI		*p*
Black	2.354	1.328	0.072	−0.253	4.961	0.077	4.956	2.408	0.152	0.228	9.683	0.040
Age (Years)	0.138	0.058	0.080	0.025	0.252	0.017	0.141	0.058	0.081	0.027	0.255	0.015
Gender	−2.294	0.842	−0.091	−3.947	−0.641	0.007	−2.317	0.842	−0.092	−3.970	−0.665	0.006
Years of Education	0.046	0.148	0.012	−0.246	0.337	0.759	0.044	0.148	0.012	−0.247	0.335	0.766
Married	1.034	1.124	0.034	−1.173	3.242	0.358	1.070	1.124	0.035	−1.136	3.277	0.341
Living Arrangement	−2.156	0.887	−0.089	−3.898	−0.414	0.015	−2.152	0.887	−0.089	−3.893	−0.410	0.016
Medicare Coverage	−0.618	0.877	−0.023	−2.341	1.104	0.481	−0.606	0.877	−0.023	−2.328	1.116	0.490
Chronic Medical Conditions (*n*)	−2.613	0.196	−0.442	−2.997	−2.228	0.000	−2.078	0.457	−0.351	−2.975	−1.182	0.000
Chronic Medical Conditions (*n*) × race/ethnicity							−0.650	0.502	−0.129	−1.636	0.335	0.196

Model 1: main effects, Model 2: main effects and interaction.

## Data Availability

Data are available upon request.
